# Porous MgNiO_2_ Chrysanthemum Flower Nanostructure Electrode for Toxic Hg^2+^ Ion Monitoring in Aquatic Media

**DOI:** 10.3390/s23187910

**Published:** 2023-09-15

**Authors:** Mohammad Imran, Eun-Bi Kim, Dong-Heui Kwak, Sadia Ameen

**Affiliations:** 1 Advanced Materials and Devices Laboratory, Department of Bio-Convergence Science, Jeonbuk National University, Jeongeup Campus, Jeongeup 56212, Republic of Korea; mohdimran@jbnu.ac.kr (M.I.); keb821@naver.com (E.-B.K.); 2 Environmental Engineering Laboratory, Department of Bioactive Material Sciences, Jeonbuk National University, Jeonju 54896, Republic of Korea

**Keywords:** MgNiO_2_, heavy metals, cyclic voltammetry, electrochemical sensor

## Abstract

A simple hydrothermal synthesis approach was used to synthesize porous MgNiO_2_ Chrysanthemum Flowers (CFs) nanostructures and applied as a sensing electrode for quick detection of hazardous mercury (Hg^2+^ ions). The morphological, structural, and electrochemical properties of MgNiO_2_ CFs were investigated. The morphological characteristic of MgNiO_2_ CFs, with a specific surface area of 45.618 m^2^/g, demonstrated strong electrochemical characteristics, including cations in different oxidation states of Ni^3+^/Ni^2+^. Using a three-electrode system for electrochemical detection, the MgNiO_2_ CFs based electrode revealed a good correlation coefficient (R^2^) of ~0.9721, a limit of detection (LOD) of ~11.7 μM, a quick response time (10 s), and a sensitivity of 8.22 μA∙μM^−1^∙cm^−2^ for Hg^2+^ ions over a broad linear range of 10–100 μM. Moreover, the selectivity for Hg^2+^ ions in tap water and drinking water was determined, and a promising stability of 25 days by MgNiO^2^ CFs electrode was exhibited. The obtained results indicate that the developed MgNiO_2_ CFs are a promising electrode for detecting hazardous Hg^2+^ ions in water and have the potential to be commercialized in the future.

## 1. Introduction

With the expansion of industry, environmental contamination has become a social concern as a result of mining for materials. Mine development seriously pollutes the aquatic environment along with soil. Additionally, industries such as chemical fertilizers, cosmetics, and gold and aluminum mining contribute significantly to heavy metal pollution in water [1]. Because heavy metal pollution is not biodegradable and may accumulate in the bodies of living beings, it is regarded as a major source of harmful environmental contamination. It causes several physical and mental health issues for humans and animals, including terrestrial and aquatic creatures [2]. Metallic elements are necessary for the human body in trace amounts, but their concentration range has a significant influence on human health. When the concentration range of a metal is below its toxic range, it is deemed safe, but when it exceeds the permissible limit, it causes a variety of cytological and physiological effects [3].

Hg^2+^ ions are the most dangerous and dominant toxicant in the environment, and ingestion of Hg^2+^ ions might damage reproductive organs, bones, brain function, kidneys, and liver, resulting in damaging the nervous system, hair, vision, and hearing loss [4,5]. Human chromosomes and genetic abnormalities are both caused by the intake of Hg^2+^ ions [6]. Hg^2+^ is a highly toxic heavy metal that might cause serious problems in aquatic environments. Mercury is a major source of environmental concern due to its stability at polluted sites and complex biological toxicity processes [7,8,9,10,11,12]. The accumulation of these metals in the body constitutes a serious hazard to human health, and therefore, the development of highly sensitive technologies for measuring trace levels of heavy metal ions has garnered considerable attention [13,14,15,16].

Many approaches for measuring heavy metals have been developed over the last few decades. Inductively coupled plasma mass spectrometry (ICP-MS), X-ray fluorescence spectroscopy (XRF), and atomic absorption spectrometry (AAS) are common techniques for assessing these metal ions [17]. However, the time-consuming method, hefty maintenance expenses, and pricey complex instruments severely limit their practical applicability. In modern society, the sensitive and selective identification of dangerous heavy metals using cost-effective and acceptable methodologies is critical [18]. Electrochemical detection techniques have recently garnered a lot of interest for heavy metal ion detection because of their capacity to detect ions with a fast analysis time, low power cost, and high sensitivity [17]. In an electrochemical approach, heavy metal ions generate changes in current, potential, electrochemical impedance, capacitance, or electrochemical luminescence that can be utilized to detect them [19].

Over the past few decades, nano-metal oxides have been widely explored in the field of electrochemical detection. Nano-metal oxides are synthesized to achieve varying sizes, stability, and morphologies. Because of these variances, these materials exhibit a wide range of electrical and photochemical characteristics, making them valuable for a wide range of applications [20]. Metal oxides, primarily transition metal oxides, have been utilized to alter electrodes for the detection of a variety of analytes [21], and just a few have been exploited for heavy metal detection [22]. Recently, secondary transition metal oxide or binary metal oxide-based materials have presented remarkable catalytic properties [23]. Apart from other metal oxides, blending MgO and NiO to form MgNiO_2_ materials has received immense attention because this combination significantly elevates the active sites for excellent catalytic reaction. In addition, various synthetic techniques can yield different catalytic impacts on electrochemical and photoelectrochemical systems. MgNiO_2_ is commonly produced using solid-state reactions or chemical approaches, such as co-precipitation, sol-gel, or hydrothermal synthesis [3]. In most reports, a solid state approach has been used to produce MgNiO_2_ materials in which high-purity magnesium oxide (MgO) and nickel oxide (NiO) powders are thoroughly mixed in the desired stoichiometric ratio at high temperature ranges from 800 °C to 1000 °C [24]. However, the hydrothermal technique involves utilizing a closed physical system and a chemical process that occurs in an aqueous solution at temperatures exceeding 100 °C to synthesize diverse chemical compounds and materials. Usually, hydrothermal synthesis offers an improved approach for obtaining small, porous, uniformly sized nanomaterials. Recently, there has been a surge in research focused on hydrothermal synthesis for secondary metal oxide materials, including MgFe_2_O_4_, MgNiO_2_, LiFePO_4_ etc. [25].

In this study, MgNiO_2_ CFs were synthesized using a simple hydrothermal approach and utilized for the three-electrode electrochemical system for the detection of toxic Hg^2+^ ions. Morphological, structural, optical, and electrochemical investigations are performed for as-synthesized MgNiO_2_ CFs, and the sensor performances in terms of sensitivity, stability, selectivity, repeatability, and detection limit for Hg^2+^ ions are thoroughly investigated. The sensing behavior of MgNiO_2_ CFs based electrode is examined by measuring the cyclicvoltametry (CV) and linear sweep voltammetry (LSV) in 0.1 M PBS (pH = 7) by varying the concentration of Hg^2+^ ions. MgNiO_2_ CFs based electrode shows a broad linear range of 1 μM^–1^ mM and a limit of detection (LOD) of ~373.9 nM with a good sensitivity of 9.008 μA∙μM^−1^∙cm^−2^ for Hg^2+^ ions.

## 2. Materials and Methods

### 2.1. Synthesis of MgNiO_2_ CFs

MgNiO_2_ was synthesized by a simple hydrothermal method. A total of 0.214 g of magnesium acetate tetrahydrate (CH_3_COO)_2_Mg∙4H_2_O, Sigma-Aldrich, St. Louis, MO, USA), 0.498 g of nickel(II) acetate (CH_3_COO)_2_Ni∙4H_2_O, Sigma-Aldrich, St. Louis, MO, USA) and 0.6 g of urea (CO(NH_2_)_2_, Sigma-Aldrich, Missouri, United States) were dissolved in 40 mL of deionized (DI) water [26]. After that, the solution was magnetically stirred at room temperature for 1 h. The mixed solution was subjected to Teflon-lined stainless steel for 10 h at 120 °C. After completion of the reaction, the product was washed with DI water and ethanol and centrifuged for 15 min at ~3000 rpm to obtain a white solid product. The product was then dried overnight at 60 °C in an oven and calcined at 650 °C for 6 h.

### 2.2. Characterization of MgNiO_2_ CFs

Field emission scanning electron microscopy (FESEM, Hitachi S-4700, Tokyo, Japan) and a transmission electron microscope (TEM, H-7650, Hitachi, Tokyo, Japan) were used to identify the morphological properties. Energy dispersive X-ray spectroscopy (EDS) was used to determine the elemental composition. To explain the crystal characteristics, X-ray diffraction (XRD, Rigaku, Woodlands, TA, USA, Cu K, = 1.54178 Å) in the Bragg angle range of 20° to 80° was used. A UV-visible spectrophotometer (JASCO, V-670) was used to measure the absorption characteristics. The structural characteristics were determined by Fourier transform infrared (FTIR, IR300, Nicolet, QC, Canada,) spectroscopy in the 400–4000 cm^−1^ region and Raman (Renishaw, Wotton-under-Edge, Old Town, UK) spectroscopy in the 200–1400 cm^−1^ ranges, respectively [26]. Surface characteristics of MgNiO_2_ CFs, such as specific surface area and pore size distribution, were investigated using the Brunauer-Emmett-Teller (BET) method with a Micromeritics Tristar 3000. The X-rays Photoelectron Spectroscopy (XPS; KRATOS AXIS-Nova, Manchester, UK) evaluated the surface composition and element states with a 0–1400 eV energy range.

### 2.3. Electrochemical Sensing of Hg^2+^ Ions Using MgNiO_2_ CFs Electrode

For the observation of Hg^2+^ ions in the solution medium, cyclic voltammetry (CV) and linear sweep voltammetry (LSV) were used. The electrolyte was prepared using 0.1 M phosphate buffered saline (PBS) at concentrations ranging from 1–100 μM Hg^2+^ ions. For electrode preparation, a cleaned 3 mm diameter screen printed electrode (SPE) was used, and a paste of 0.05 wt% of MgNiO_2_ powder with nafion solution was deposited on the SPE surface by the doctor blade method [27,28,29,30]. Thereafter, the nafion binder was subsequently removed by annealing SPE for 15 min at 80 °C in an oven. Three electrodes with SPE as working electrode, Ag/AgCl reference electrode, and a gold wire counter electrode were used to detect Hg^2+^ ions. For cyclic voltammetry (CV) measurements, the scan rate was fixed at 10 mV/s using different concentration of Hg^2+^ ions. The sensitivity of the electrochemical sensor is estimated by dividing the slope of the calibrated plot by the active area of the sensing electrode. The limit of detection (LOD) was calculated from the following equation:

LOD = 3.3 × SD/slope(1)

where SD = standard deviation

## 3. Results

### 3.1. Morphological Properties and Element Analysis of MgNiO_2_ CFs

**Figure 1:**
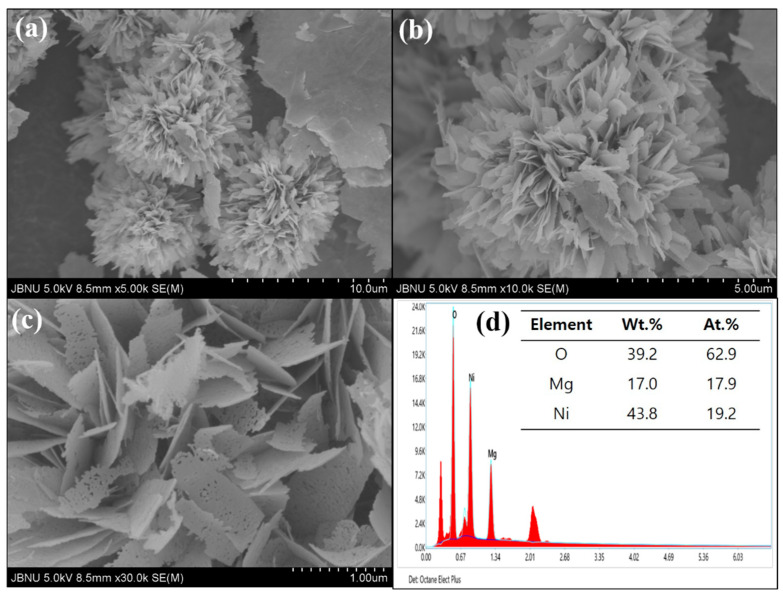
FESEM images at low magnification (**a**,**b**), high magnification (**c**), and EDX spectrum (**d**) of MgNiO_2_ CFs.

FESEM was employed to investigate the morphology of MgNiO_2_ nanostructures, as shown in Figure 1a–c. At low magnification, the synthesized MgNiO_2_ exhibited the morphology of Chrysanthemum Flowers (CFs) with an average diameter of ~6.34 μm, as shown in Figure 1a,b. Figure 1c exhibits the MgNiO_2_ CFs at high magnification with thin sheet aggregation and an average thickness of ~25 nm. The existence of several pores in the sheet was apparent, suggesting that the synthesized MgNiO_2_ CFs are porous and have a high specific surface area. Additionally, SEM-EDS was used to verify the purity of the synthesized MgNiO_2_ CFs. The EDS spectra of MgNiO_2_ CFs are shown in Figure 1d. Herein, no peaks other than Mg, Ni, and O were seen, suggesting that the synthesized MgNiO_2_ CFs are pristine. The traces of Pt peaks can be seen in an EDS image, which might be due to the surface coating of Pt during FESEM. Furthermore, the atomic percentages (at %) of Mg, Ni, and O were 17.9%, 19.2%, and 62.9%, respectively, with a comparatively high proportion of oxygen perhaps owing to oxygen or moisture in the air sticking to the MgNiO_2_ CFs. The FESEM results confirmed that the synthesized MgNiO_2_ was pure with a unique Chrysanthemum Flower structure.

**Figure 2:**
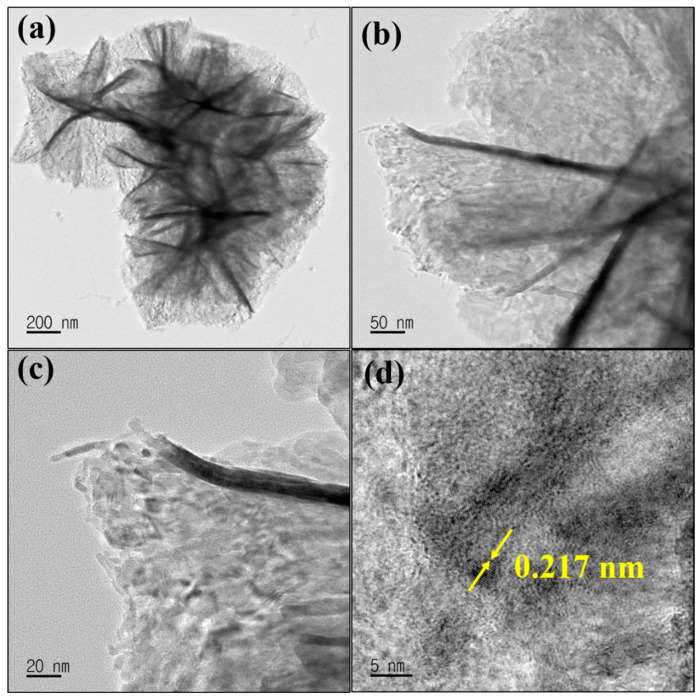
HRTEM images at low magnification (**a**–**c**) and high magnification (**d**) of MgNiO_2_ CFs.

TEM and HRTEM were used to confirm the morphology of MgNiO_2_ CFs. The TEM image in Figure 2a corresponds with FESEM results, exhibiting the size of MgNiO_2_ CFs as ~5–7 μm. Figure 2b,c is a high magnification TEM image that reveals a clear lattice structure, showing that the synthesized MgNiO_2_ CFs have an excellent crystalline phase. The HRTEM image, as shown in Figure 2d, exhibited a lattice distance of ~0.217 nm, which corresponds to the (200) crystal plane [31,32].

### 3.2. Structural and Surface Properties of MgNiO_2_ CFs

**Figure 3:**
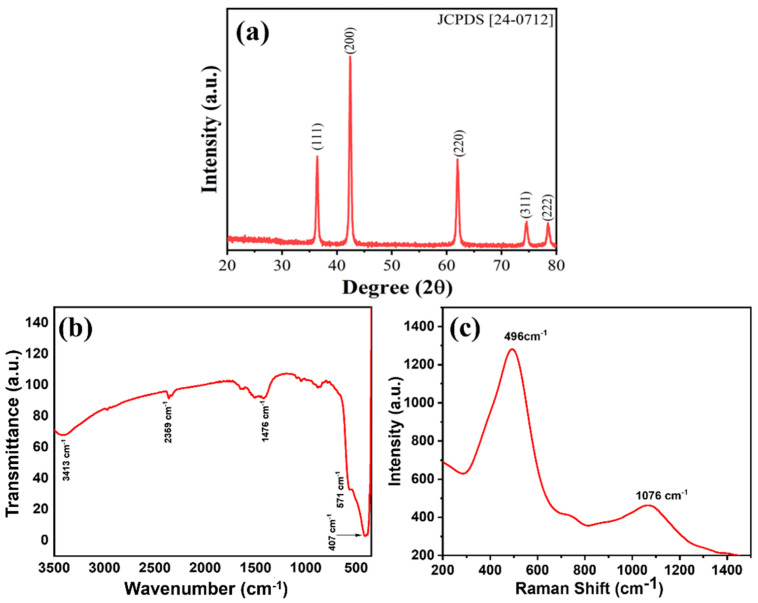
XRD patterns (**a**) FTIR spectroscopy (**b**) Raman spectroscopy (**c**) of MgNiO_2_ CFs.

The crystalline characteristics of MgNiO_2_ CFs were identified by X-ray diffraction (XRD), as shown in Figure 3a. The observed diffraction peaks at ~36.40° (111), ~42.40° (200), ~61.98° (220), ~74.58° (311), and 78.52° (222) correspond to typical MgNiO_2_ (JCPDS 24-0712). A prominent diffraction peak appeared at ~42.40°, indicating that MgNiO_2_ CFs had primarily grown in the (200) plane. The Scherrer equation [33] was used for calculating the size of the crystals of MgNiO_2_ CFs:

(2) $$\tau =\left(\frac{k\lambda }{\beta cos\theta }\right)$$

where τ is the crystal size, *β* is the full width at half maximum (FWHM), *θ* is the Bragg angle, *k* is the Scherrer constant (crystal shape = 0.94), and *λ* is the X-ray wavelength (Cu = 1.54178 Å). Herein, the strongest lattice plane of ~42.4° (200) is chosen, and an FWHM (*β*) value of 0.3983° and a crystal size (τ) of ~22.35 nm is obtained. This is similar to the thickness of a single sheet, as shown in FESEM, indicating that the MgNiO_2_ sheets are agglomerated into a single layer crystal structure. In addition, MgNiO_2_ CFs showed no additional peaks, confirming the purity of the synthesized nanostructures. FTIR analysis was employed to characterize the structural properties of the MgNiO_2_ CFs, as depicted in Figure 3b. The O–H stretching vibration of water molecules was adsorbed on the surface causes the appearance of a common wide peak at ~3413 cm^−1^. The Mg–O vibration coupling frequency was involved in the existence of a peak at ~571 cm^−1^, whereas the Ni–O vibration coupling frequency was confirmed by the peak at ~407 cm^−1^. The observed peaks in MgNiO_2_ CFs matched well with the reported values in the published literature [34,35]. Furthermore, quite weak bands were observed at ~2359 cm^−1^ and ~1476 cm^−1^, corresponding to atmospheric CO_2_ adsorption [36]. The Raman spectroscopy observations of MgNiO_2_ CFs are presented in Figure 3c. The peak at ~496 cm^−1^ was attributed to a single phonon (1P) TO mode, and the peak at ~1076 cm^−1^ was assumed to be the result of LO modes. The existence of NiO was the primary cause for the observed Raman peaks [37]. According to the Raman spectra, the synthesized MgNiO_2_ CFs showed high phase purity.

**Figure 4:**
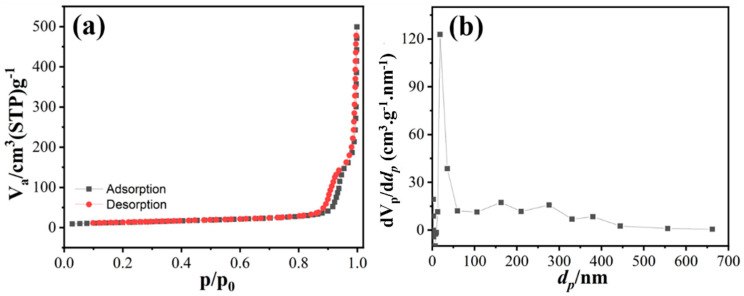
N_2_ adsorption and desorption plot (**a**) pore size contribution (**b**) of MgNiO_2_ CFs.

Figure 4 exhibits the adsorption/desorption of N_2_ gas via Brunauer-Emmett-Teller (BET) analysis of MgNiO_2_ CFs. As shown in Figure 4a, the overall specific surface area of MgNiO_2_ CFs was ~45.618 m^2^/g, with voids providing the total specific surface area at ~41.041 m^2^/g. Herein, because of the high porosity of MgNiO_2_ CFs, the specific surface area increased significantly. Figure 4b shows the average diameter of the pores as ~61.871 nm. The results showed a match with the morphological properties of FESEM results, implying that the increased surface area of MgNiO_2_ CFs might enhance the active sites for the detection of target heavy metals, resulting in enhanced sensitivity.

**Figure 5:**
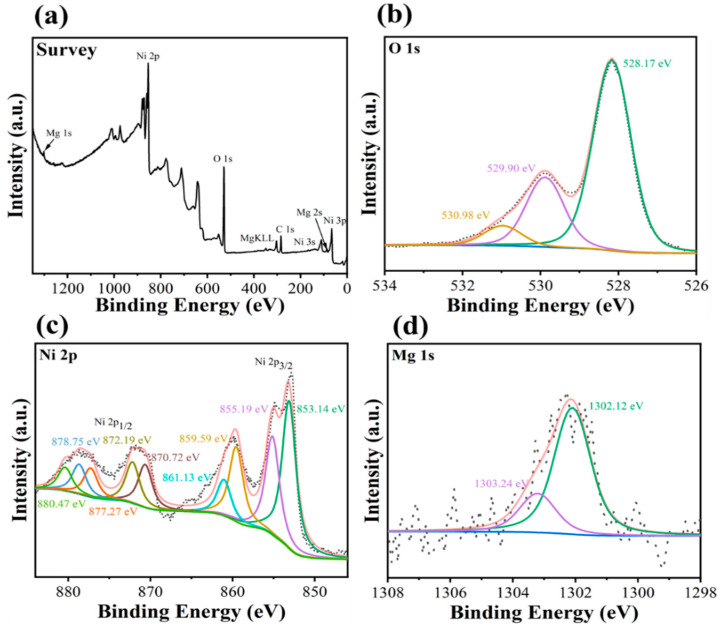
XPS spectra of MgNiO_2_ CFs (**a**) survey profile, (**b**) O ls, (**c**) Ni 2p (**d**) Mg 1s.

X-ray photoelectron (XPS) analysis was utilized to determine the surface binding energies of MgNiO_2_ CFs. Figure 5a shows the survey profile, which exhibits distinct peaks of O 1s, Ni 2p, and Mg 1s, indicating the existence of Ni, Mg, and O in MgNiO_2_ CFs. Figure 5b depicts a high-resolution O 1s spectra with peaks at ~528.17 eV, ~529.90 eV, and ~530.98 eV resulting from oxygen bonding with Ni^2+^, Mg^2+^, and Ni^3+^ ions, respectively [38,39]. It suggests that the MgNiO_2_ CFs bonding is composed of three ions: Ni^2+^, Mg^2+^, and Ni^3+^. In Figure 5c, the high-resolution spectrum of Ni 2p depicts the peaks at ~853.14 eV and 855.19 eV, which might be due to Ni 2p_3/2_ of Ni^2+^ and Ni^3+^, while the peaks at ~870.72 eV and ~872.19 eV are due to Ni 2p_1/2_ of Ni^2+^ and Ni^3+^. The MgNiO_2_ CFs were assumed to be in the Ni^3+^ and Ni^2+^ states [40]. The peaks at ~859.59 eV and ~861.13 eV were Ni 2p_3/2_ satellites, whereas the peaks at ~877.27 eV, ~878.75 eV, and ~880.47 eV were Ni 2p_1/2_ satellites. Furthermore, the difference between the Ni 2p_3/2_ and Ni 2p_1/2_ double peaks was ~17.58 eV, indicating the existence of various oxidized Ni^3+^ and Ni^2+^ ions [41,42]. Figure 5d shows the high-resolution Mg 1s spectra, which has binding energies of ~1302.12 eV for Mg and ~1303.24 eV for Mg–O [23]. Thus, the existence of Ni^3+^, Ni^2+^, and Mg^2+^ ions was explained by the XPS spectra of MgNiO_2_ CFs.

### 3.3. Optical Properties of MgNiO_2_ CFs

**Figure 6:**
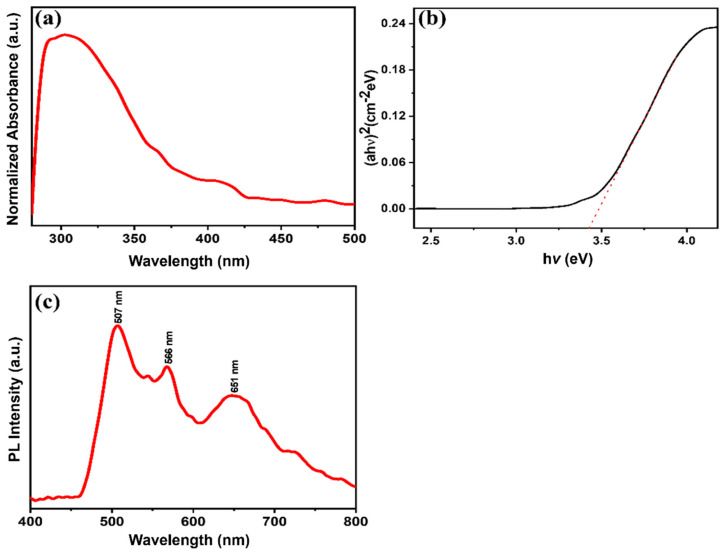
UV-vis (**a**), calculated optical band (αhν)^2^ vs. hν (**b**) (Dotted red line shows the intercept of plot) and PL spectrum (**c**) of MgNiO_2_ CFs.

UV-vis in the range of 300–800 nm was applied to investigate the optical characteristics of the MgNiO_2_ CFs. The absorption peak of MgNiO_2_ CFs at ~303 nm is shown in Figure 6a. The optical band gap of MgNiO_2_ CFs was calculated using the Tau Equation (2) based on the UV-vis graph [43].

(αhν)^n^ = A (hν − E_g_) (3)

where α is the absorption coefficient, hν is photon energy, A is absorbance, E_g_ is optical band gap, and n is a number (n = 2) describing the transition process. The optical band gap of ~3.41 eV was determined by (αhν)^2^ versus hν energy, as shown in Figure 6b. This value was lower than the optical bandgap of conventional NiO (~3.6 eV), implying that MgNiO_2_ CFs can be excited more easily than typical NiO [44]. The photoluminescence emission spectra of MgNiO_2_ CFs were measured in the 400–800 nm region at room temperature. Three emission peaks appear at ~507 nm, ~566 nm, and ~651 nm. Herein, the peaks at ~507 nm and ~566 nm were related to NiO, and the PL peaks were upshifted by a smaller particle size and appeared as a double peak at ~20 nm [45]. These findings matched well to the crystal size determined by XRD (~25 nm). MgO is shown by the peak at ~651 nm [46]. Herein, the photoluminescence peak was caused by an electronic shift involving 3d_8_ electrons of Ni^2+^ ions [47]. Direct recombination between electrons in the conduction band and holes in the valence band induced the PL spectrum to consist of strong and wide peaks [48].

### 3.4. Sensing Performance, Selectivity, and Real Sample Performance of MgNiO_2_ CFs

**Figure 7:**
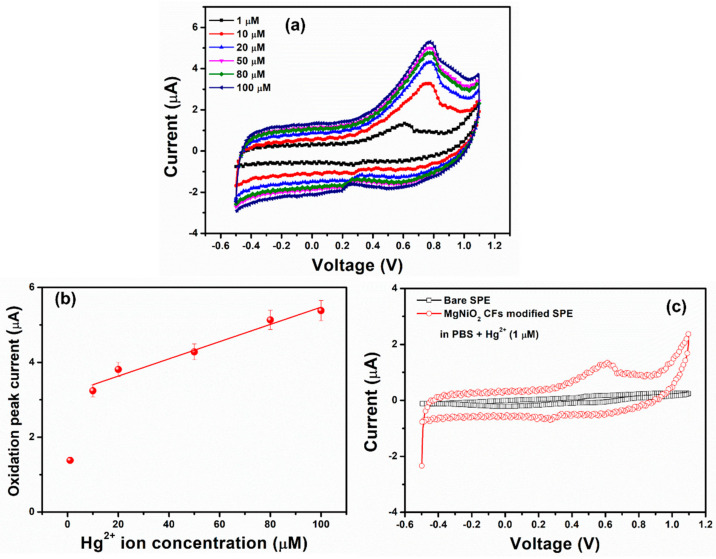
CV plot (**a**), calibrated oxidation current versus concentration of Hg^2+^ ions (**b**), and I–V curves with varying Hg^2+^ ion concentrations from 1 μM~100 μM concentration of the Hg^2+^ ion in 0.1 M PBS and (**c**) CV plots of bare SPE and MgNiO_2_ CFs modified electrode in PBS with Hg^2+^ (μM).

The electrochemical characteristics of the MgNiO_2_ CFs modified electrode towards the detection of Hg^2+^ ions were investigated using cyclicvoltammetry (CV). A three-electrode system was utilized with MgNiO_2_ CFs as the working electrode, Ag/AgCl as the counter electrode, and Pt as the reference electrode [49]. The target electrolyte was prepared by dissolving different concentration of Hg^2+^ ions (1–100 μM) in phosphate buffer solution (PBS, pH = 7.0), and the CV plots were measured at a scan rate of 50 mV/s, as shown in Figure 7a. In our work, as the concentration of Hg^2+^ ion increased, the oxidation or reduction current increased correspondingly. The maximum oxidation current peak of ~5.20 μA was observed for 100 μM of Hg^2+^ ions, which was 3 times higher than the oxidation current for 1 μM. Notably, a higher oxidation peak suggested a quicker electron transfer process in the electrochemical system and a stronger electrocatalytic behavior of the electrode [31]. The excellent and rapid sensing response of the MgNiO_2_ CFs electrode for Hg^2+^ ions might be related to the conductive character of Ni^3+^/Ni^2+^ in the charge transfer process [50]. Due to excellent electrocatalytic efficiency towards Hg^2+^ ions, the MgNiO_2_ CFs electrode exhibited an increase in anodic current. In order to calculate the sensitivity of the MgNiO_2_ CFs electrode, the oxidation current versus Hg^2+^ ions concentration is displayed in Figure 7b. The fabricated electrode showed a correlation coefficient (R^2^) of ~0.9721, a limit of detection (LOD) of ~11.7 μM, a constant sensitivity of ~8.22 μA∙μM^−1^∙cm^−2^, and high linearity in the 10–100 μM wide range. The existence of significantly promising sensitivity might be due to the large surface area of MgNiO_2_ CFs, which allows considerable analytic adsorption on the electrode surface [21]. The detection of Hg^2+^ ion as reported by other workers is discussed in Table 1. In comparison, MgNiO_2_ CFs based electrode displayed a low LOD and a high sensitivity across a large linear range towards the detection of the Hg^2+^ ion.

To investigate the effect of MgNiO_2_ on sensing behavior, CV measurements of Hg^2+^ ions in 0.1 M PBS electrolyte on bare SPE and MgNiO_2_ CFs modified SPE were performed. According to Figure 7c, the bare SPE posed the least current response to 1 μM concentration of Hg^2+^ ions in PBS electrolyte, whereas a prominent current response to 1 μM concentration of Hg^2+^ ions was recorded by MgNiO_2_ CF-modified SPE. This noted change in current response indicates the sensing behavior of MgNiO_2_ CFs toward Hg^2+^ ions at very low concentrations. Therefore, the obtained result showed strong electrocatalytic characteristics of MgNiO_2_ CFs electrode to sense Hg^2+^ ions at low traces.

**Figure 8:**
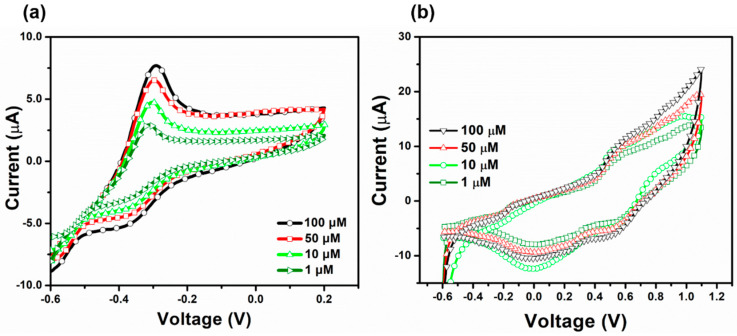
CV plots for (**a**) Cr^3+^ and (**b**) Cu^2+^ ions in 0.1 M PBS of MgNiO_2_ CFs modified electrode.

To investigate the electrode selectivity, the MgNiO_2_ CFs electrode were tested by electrochemical method for the detection of other heavy metal ions, such as Cr^3+^ and Cu^2+^. The PBS electrolytes of Cr^3+^ and Cu^2+^ ions were prepared in the same way as the Hg^2+^ ions. The MgNiO_2_ CFs electrode was measured by CV investigation at a constant scan rate of 50 mV/s. Figure 8a depicts that the oxidation current increases with increasing Cr^3+^ ion concentration. The result was comparable to Hg^2+^, but the oxidation peak emerged at a lower voltage range, implying that Cr^3+^ might be evaluated independently of Hg^2+^. The CV curve of Cu^2+^ in Figure 8b revealed erratic results regardless of concentration rise, indicating that the MgNiO_2_ CFs electrode has no selectivity for Cu^2+^.

**Figure 9:**
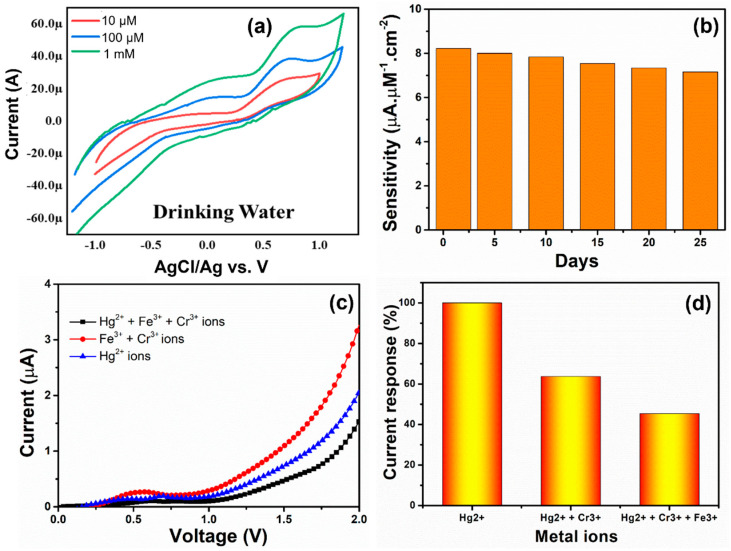
(**a**) CV plots for real samples sensing in drinking water, and (**b**) stability test of MgNiO_2_ CFs modified electrode. (**c**) I–V curves and (**d**) current response (%) of MgNiO_2_ CFs modified electrode with interfering metal ions in PBS electrolyte.

The Hg^2+^ ions sensing behavior in drinking water was tested to determine the actual usage of the MgNiO_2_ CFs electrode. The CV plots of Hg^2+^ ion in drinking water are shown in Figure 9a, exhibiting increasing concentrations of Hg^2+^ ion, and higher voltage values. This confirms the actual usability of the MgNiO_2_ CFs electrode. Moreover, the shapes of CV curves differed from almost similar oxidation peaks (as observed in Figure 7a), which might be related to direct testing in drinking water. To evaluate the stability of the MgNiO_2_ CFs electrode, as shown in Figure 9c, the sensitivity performances for Hg^2+^ ions were measured at regular intervals over a period of 25 days. The I–V properties of the MgNiO_2_ CFs electrode were examined after every 5-days, and the I–V current values for the detection of Hg^2+^ ions remained at roughly ~86% of the initial value without exhibiting a significant drop in the performance. This demonstrates an excellent stability of the MgNiO_2_ CFs electrode toward the detection of Hg^2+^ ions. The obtained stability results clearly showed the long-term viability of the MgNiO_2_ CFs electrode towards electrochemical sensing for the detection of harmful Hg^2+^ ions.

The selectivity of the MgNiO_2_ CFs electrode toward Hg^2+^ ions was further examined by measuring the current response in PBS electrolytes with Hg^2+^ ions (10 µM) and mixtures with other metal ions. As shown in Figure 9c, the current responses were considerably decreased when Fe^3+^ (10 µM) and Cr^3+^ (10 µM) ions were mixed with Hg^2+^ ions. In other words, interfering species such as Fe^3+^ (10 µM) and Cr^3+^ (10 µM) ions and Hg^2+^ ions in PBS electrolytes resulted in lowering the current response compared to a high current response observed only by Hg^2+^ ions. Figure 9d displays the percentage response of interfering metal ions by the MgNiO_2_ CFs electrode. With the MgNiO_2_ CFs electrode, ~100% sensitivity of Hg^2+^ ions was achieved, whereas the sensing responses were lowered towards Cr^3+^ + Fe^3+^ ions (~62%) and Cr^3+^ + Fe^3+^ + Hg^2+^ ions (~44%). Thus, the MgNiO_2_ CFs electrode expressed good selectivity for Hg^2+^ ions compared to other heavy metal ions.

## 4. Conclusions

A simple hydrothermal technique was used to synthesize MgNiO_2_ nanomaterials in the form of Chrysanthemum Flowers (CFs). The synthesized MgNiO_2_ CFs showed significant size pores of ~61.871 nm with a high specific surface area of ~45.618 m^2^/g, which created an extensive sensing active site. The Ni ions in the MgNiO_2_ CFs showed multiple Ni^3+^/Ni^2+^ oxidation states, which supported good conductive characteristics and promising electronic behavior. The MgNiO_2_ CFs electrode had a high correlation coefficient (R^2^) of ~0.9721, a low limit of detection (LOD) of ~11.7 μM, a fast reaction time (10 s), and a sensitivity of ~8.22 μA∙μM^−1^∙cm^−2^ towards the detection of Hg^2+^ ions over a wide linear range of 10–100 μM. Furthermore, the MgNiO_2_ CFs electrode was tested to detect other heavy metal ions, namely Cr^3+^ and Cr^2+^, and the obtained results confirmed that the MgNiO_2_ CFs electrode is a promising material for the detection of Hg^2+^ ions and could be utilized in the future for testing other toxic materials.

## Figures and Tables

**Table 1:** Comparison of different analytes and methods for the detection of the Hg^2+^ ion. Correct the reference in the table as [47,48,49].

Material and Method	LOD (μM)	Sensitivity (μA.μM^−1^cm^−2^)	Linear Range (μM)	R^2^	Ref.
AuNPs-GCE (Electrodeposition method)	0.64	0.274	-	-	[51]
AuNPs (Turkevich method)	1.0	0.269	0.36–10	-	[52]
Au-TiO_2_ (Sol-gel method)	1.0	-	0.05–4	-	[53]
MgNiO_2_ (Hydrothermal synthesis)	11.7	8.22	10–100	0.9721	This work

## Data Availability

Not applicable.
